# Association between household secondhand tobacco smoke exposure and regular e-cigarette use among adolescents: evidence from a national school-based survey

**DOI:** 10.1093/eurpub/ckaf150

**Published:** 2025-08-24

**Authors:** Yusuff Adebayo Adebisi, Najim Z Alshahrani, Abdulrakib Abdulrahim, Don Eliseo Lucero-Prisno

**Affiliations:** College of Social Sciences, University of Glasgow, Glasgow, United Kingdom; Department of Family and Community Medicine, Faculty of Medicine, University of Jeddah, Jeddah, Saudi Arabia; Department of Medical Microbiology, Faculty of Medicine and Health Sciences, Universiti Putra Malaysia, Serdang, Malaysia; Department of Global Health and Development, London School of Hygiene and Tropical Medicine, London, United Kingdom; Center for University Research, University of Makati, Makati City, Philippines; Research Office, Palompon Institute of Technology, Palompon, Leyte, Philippines

## Abstract

Adolescent e-cigarette use is of public health interest in England, yet the influence of household environmental factors remains poorly understood. This cross-sectional study examined the association between secondhand tobacco smoke exposure in the home and regular e-cigarette use among adolescents. We analysed data from 12 297 adolescents aged 11–15 years who participated in the 2023 wave of the Smoking, Drinking and Drug Use among Young People in England (SDD) survey. The primary exposure was self-reported frequency of secondhand smoke exposure in the home or someone else’s home, categorized into five levels. The outcome was regular e-cigarette use, defined as vaping at least once per week. Logistic regression models estimated crude and adjusted associations, controlling for age, gender identity, ethnicity, family affluence, and household or peer smoking. Regular e-cigarette use was reported by 5.8% (*n* = 716) of the sample. Compared to those never exposed, adolescents exposed to secondhand smoke ‘daily or most days’ had significantly higher odds of regular e-cigarette use [adjusted odds ratio (aOR) = 7.25; 95% confidence interval (CI): 5.62–9.34; *P < .*001]. A clear dose–response relationship was observed across exposure categories (*P* for trend <.001), with increasing exposure linked to progressively higher odds of vaping. Among adolescents who had never smoked cigarettes, daily or near‑daily exposure to household secondhand smoke was still strongly associated with regular e‑cigarette use (aOR = 5.04; 95% CI: 3.64–6.99; *P < .*001). Frequent secondhand tobacco smoke exposure in the home is a strong and independent correlate of regular e-cigarette use among adolescents in England.

## Introduction

E-cigarette use among adolescents has risen sharply in recent years, prompting concern from researchers, educators, and policymakers worldwide [1, 2]. In England, although conventional cigarette smoking among young people has declined, vaping has rapidly emerged as a common behaviour [3, 4]. National survey data indicate that a growing proportion of adolescents are experimenting with or regularly using e-cigarettes, despite regulations aimed at restricting youth access and marketing [5]. This trend raises critical questions about the drivers of e-cigarette uptake and the environments that may facilitate or discourage its use.

Previous studies have identified several influences on adolescent vaping, including peer use, curiosity, risk perception, exposure to online marketing, and access to flavoured products [6–8]. However, the influence of the home environment, particularly exposure to secondhand tobacco smoke, has been underexplored. While public health messaging has long emphasized the physical harms of secondhand smoke, its psychological and behavioural effects, especially its potential to normalize nicotine use, remain understudied. Adolescents exposed to smoking in the home may perceive tobacco and related products, such as e-cigarettes, as acceptable or commonplace. Observational social learning theory suggests that young people may model behaviours observed in parents or family members [9], potentially extending to the uptake of nicotine through vaping.

Although some studies have explored associations between parental smoking and adolescent vaping [10–12], few have specifically examined the frequency of secondhand smoke exposure or its relationship with regular e-cigarette use. Moreover, existing research often fails to account for adolescents’ smoking status, leaving unclear whether secondhand smoke exposure is merely a proxy for cigarette use or an independent predictor for vaping initiation. Clarifying this distinction is essential for effective prevention efforts, as it could reveal whether current policies, which primarily target youth access and peer influence, overlook a critical modifiable factor within the household.

This study investigates the association between household secondhand smoke exposure and regular e-cigarette use among secondary school students in England. Using data from the 2023 wave of the nationally representative Smoking, Drinking and Drug Use among Young People in England (SDD) survey, we examine the dose–response relationship of secondhand smoke exposure with adolescent vaping. We also assess whether this association persists among never-smokers to better understand its independent effect.

## Method

### Study design

This cross-sectional study investigated the association between secondhand smoke exposure in the home and regular e-cigarette use among adolescents in England. Data were drawn from the 2023 wave of the SDD survey, a nationally representative school-based study conducted by the National Centre for Social Research on behalf of National Health Service (NHS) Digital [13]. The SDD survey monitors key health behaviours among pupils aged 11–15 and includes questions on tobacco, e-cigarettes, alcohol, drugs, mental health, and social influences. The questionnaire was completed online by pupils in classroom settings during school hours. Its anonymous, self-administered format is designed to maximize honest disclosure and reduce social desirability bias.

### Data source and study population

The sample was drawn from pupils in school years 7–11 (typically aged 11–15) across 185 schools in England [13]. The full dataset comprised 17 466 respondents from both core sample schools (*n* = 13 387) and schools that volunteered to participate (*n* = 4079). The survey used a two-stage stratified sampling design. In the first stage, schools were selected within each region based on school type and local area deprivation. In the second stage, three mixed-ability classes were randomly selected from each school—two from years 9–11 and one from years 7 or 8. The survey was conducted during a single school period, with pupils completing the questionnaire independently and anonymously. We excluded 5169 respondents with missing data on the main exposure (secondhand smoke exposure) or the outcome (regular e-cigarette use), resulting in a final analytic sample of 12 297 adolescents with complete data on both variables.

### Measures

The primary exposure variable was the frequency of exposure to secondhand smoke in the home or someone else’s home. Pupils were asked: ‘In the past year, how often were you in the same room as someone smoking (either inside your home or inside someone else’s home)?’ Response options included: (i) every day or most days, (ii) once or twice a week, (iii) once or twice a month, (iv) less often than once a month, (v) never in the past year, (vi) do not know, and (vii) prefer not to say. For analysis, responses were grouped into five categories: every day or most days, once or twice a week, once or twice a month, less often than once a month, and never in the past year. Pupils who selected ‘do not know’ or ‘prefer not to say’ were excluded. The survey item was designed to capture overall secondhand smoke exposure in private indoor environments, regardless of whether it occurred in the pupil’s own home or someone else’s home. These contexts were combined because both represent settings where adolescents may have limited control over smoking behaviours and where exposure can influence normative attitudes.

The primary outcome was regular e-cigarette use, defined as using e-cigarettes at least once a week. This was based on the question: ‘Read the following statements carefully and tick the box next to the one which best describes you. Think about times when you may have had a puff or two as well as using whole vapes’. Response options were: (i) I have never tried vapes, (ii) I have used vapes only once or twice, (iii) I used to use vapes, but I do not now, (iv) I sometimes use vapes, but I do not use them every week, (v) I use vapes regularly, once a week or more, (vi) do not know, and (vii) prefer not to say. Pupils who selected ‘do not know’ or ‘prefer not to say’ were also excluded. Responses were recoded into a binary variable, with regular weekly users (option 5) coded as 1 and all other responses coded as 0.

Covariates were selected *a priori* based on theoretical relevance and previous empirical evidence [14–16]. These included age (grouped by school year from 11 to 15), gender identity (boy, girl, another gender identity, or prefer not to say), and ethnicity (White, Mixed, Asian, Black, Other, or Missing). We also included household or peer smoking status, based on whether anyone close to the respondent smokes cigarettes. Socioeconomic status was measured using the Family Affluence Scale, which was categorized into low (<7), medium (7–10), and high affluence (11+) bands. To preserve sample size, missing responses on any covariate were coded as a separate category.

### Statistical analysis

We first described the sample by summarizing the distribution of demographic and household characteristics, stratified by regular e-cigarette use status. Frequencies and percentages were calculated for all categorical variables, and group differences were assessed using Pearson chi-squared tests. We then used multivariable logistic regression to examine the association between secondhand smoke exposure and regular e-cigarette use. In the primary model, secondhand smoke exposure was entered as a five-category variable: never in the past year (reference), less than monthly, once or twice per month, once or twice per week, and daily or most days. The model adjusted for age, gender identity, ethnicity, household or peer smoking, and socioeconomic status, measured using the Family Affluence Scale. Odds ratios (ORs), 95% confidence intervals (CIs), and *P* values were reported. Standard errors were adjusted for clustering at the school level using robust variance estimation. To further examine whether there was a linear trend in the association between secondhand smoke exposure and vaping, we repeated the analysis treating exposure as an ordinal variable ranging from 1 (never) to 5 (daily or most days). This dose–response model tested whether increasing levels of exposure were associated with progressively higher odds of regular e-cigarette use, while adjusting for the same covariates.

To explore whether the association was independent of cigarette smoking, we conducted a stratified analysis restricted to adolescents who reported never smoking. This allowed us to evaluate the association between secondhand smoke exposure and regular e-cigarette use in a population naïve to tobacco. All analyses were performed using Stata version 18, with *P* values <.05 considered statistically significant.

## Result

Table 1 presents the characteristics of the 12 297 adolescents included in the analytic sample, stratified by regular and non-regular e-cigarette use. Regular users (*n* = 716) were significantly older than non-regular users (*n* = 11 581), with nearly half (45.0%) aged 15 years, compared to 18.3% among non-regular users (*P < .*001). Among regular e-cigarette users, 52.1% were girls, 35.0% were boys, and 9.4% identified as another gender (*P* < .001). While the majority of participants identified as White across both groups, regular users were more likely to be White (73.9%) and less likely to be Asian or Black compared to non-regular users (*P < .*001). Adolescents from families with medium or high affluence were more likely to report regular use, although the proportion of missing responses on affluence was higher among regular users (29.3%) than non-regular users (14.4%) (*P < .*001). Exposure to smoking within the household or peer group was markedly more common among regular users (89.1%) than among non-regular users (67.0%) (*P < .*001). All observed group differences were statistically significant.

**Table 1.** Characteristics of adolescents stratified by regular and non-regular e-cigarette use^a^

Characteristics	Nonregular e-cigarette user (*n* = 11 581)	Regular e-cigarette user (*n* = 716)	All (*n* = 12 297)	*P*
Age, *n* (%)				<.001
11	1484 (12.8)	33 (4.6)	1517 (12.3)	
12	2617 (22.6)	44 (6.2)	2661 (21.6)	
13	2904 (25.1)	135 (18.9)	3039 (24.7)	
14	2454 (21.2)	182 (25.3)	2636 (21.4)	
15	2122 (18.3)	322 (45.0)	2444 (19.9)	
Sex, *n* (%)				<.001
Boy	5673 (49.0)	251 (35.0)	5924 (48.1)	
Girl	5441 (47.0)	373 (52.1)	5814 (47.3)	
Other	274 (2.4)	67 (9.4)	341 (2.8)	
Prefer not to say	193 (1.6)	25 (3.5)	218 (1.8)	
Ethnicity, *n* (%)				<.001
White	7734 (66.8)	529 (73.9)	8263 (67.2)	
Mixed	655 (5.7)	39 (5.5)	694 (5.6)	
Asian	1335 (11.5)	39 (5.5)	1374 (11.2)	
Black	894 (7.7)	40 (5.6)	934 (7.6)	
Other	163 (1.4)	11 (1.5)	174 (1.4)	
Missing	800 (6.9)	58 (8.1)	858 (7.0)	
Family affluence, *n* (%)				<.001
Low affluence	1573 (13.6)	85 (11.9)	1658 (13.5)	
Medium affluence	5153 (44.5)	255 (35.6)	5408 (44.0)	
High affluence	3192 (27.6)	166 (23.2)	3358 (27.3)	
Missing	1663 (14.4)	210 (29.3)	1873 (15.2)	
Household or peer smokes, *n* (%)				<.001
No	3765 (32.5)	73 (10.2)	3838 (31.2)	
Yes	7753 (67.0)	638 (89.1)	8391 (68.2)	
Missing	63 (0.5)	5 (0.7)	68 (0.6)	

a Regular use defined as vaping at least once per week. Non-regular use includes never, former, and occasional users.

**Figure 1.**
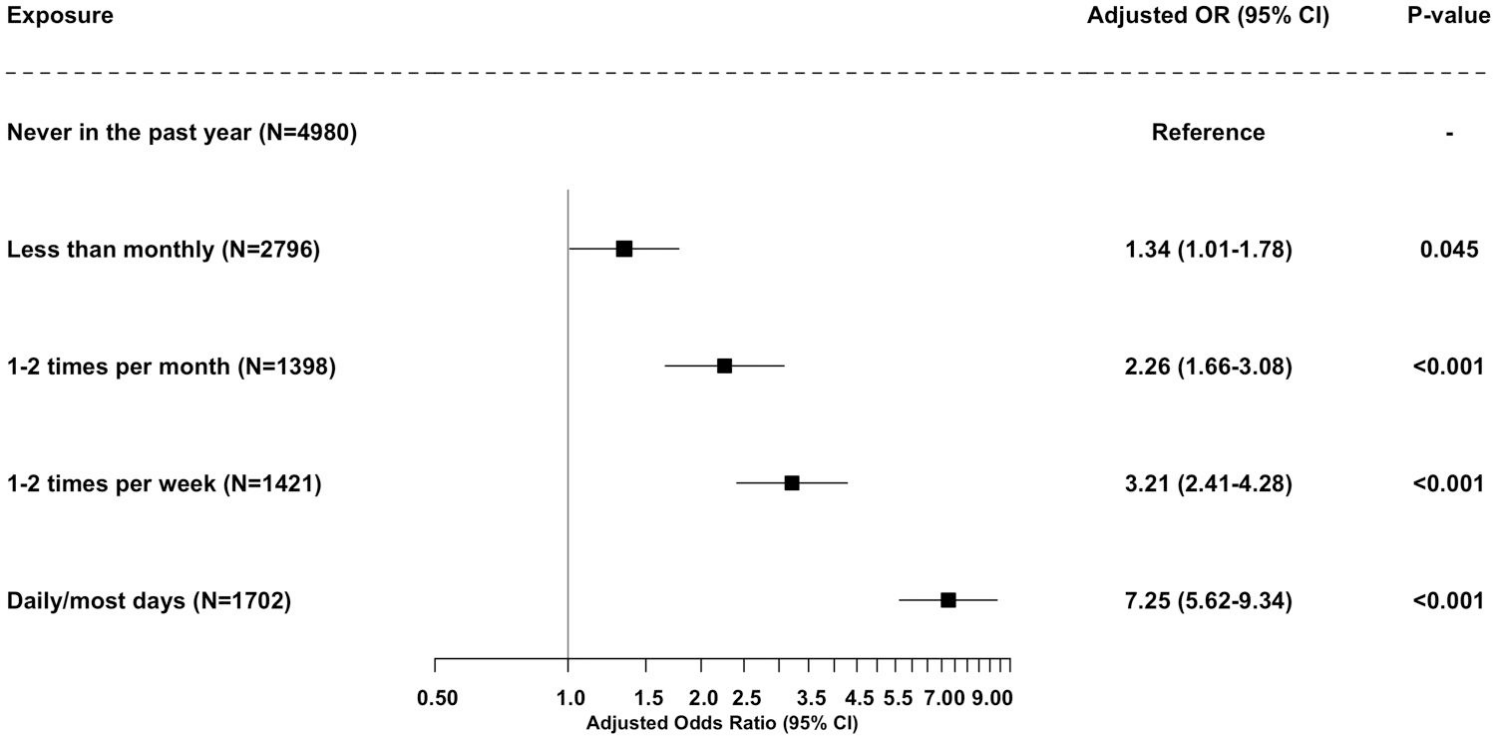
Adjusted odds ratios for regular e-cigarette use by frequency of household secondhand smoke exposure among adolescents (*n* = 12 297).

Table 2 and Fig. 1 display the crude and adjusted associations between frequency of household secondhand smoke exposure and regular e-cigarette use among all adolescents (*n* = 12 297). In the fully adjusted model, controlling for age, gender identity, ethnicity, household or peer smoking, and family affluence, there was a clear dose–response relationship between increasing exposure to secondhand smoke and the odds of regular e-cigarette use (*P* for trend <.001). Compared with adolescents who reported no exposure in the past year, those exposed less than monthly had 34% higher odds of regular e-cigarette use [adjusted OR (aOR) = 1.34, 95% CI: 1.01–1.78, *P* = .045], while those exposed 1–2 times per month had more than twice the odds (aOR = 2.26, 95% CI: 1.66–3.08, *P < .*001). Exposure 1–2 times per week was associated with over a threefold increase (aOR = 3.21, 95% CI: 2.41–4.28, *P < .*001), and daily or near-daily exposure was associated with a more than sevenfold increase in odds (aOR = 7.25, 95% CI: 5.62–9.34, *P < .*001). The ordinal trend analysis confirmed a strong, graded association across increasing levels of exposure (aOR per category = 1.66, 95% CI: 1.57–1.76, *P < .*001).

**Table 2.** Crude and adjusted associations between household secondhand smoke exposure frequency and regular e-cigarette use among all adolescents (*n* = 12 297)

Secondhand smoke exposure	*N*	Crude OR, 95% Cl, *P* value	Adjusted OR, 95% Cl, *P* value^a^
Never in the past year	4980	Reference	Reference
Less than monthly	2796	1.88 (1.43–2.48), *P < .*001	1.34 (1.01–1.78), *P* = .045
1–2 times per month	1398	3.10 (2.31–4.16), *P < .*001	2.26 (1.66–3.08), *P < .*001
1–2 times per week	1421	4.37 (3.33–5.73), *P < .*001	3.21 (2.41–4.28), *P < .*001
Daily/most days	1702	10.40 (8.25–13.12), *P < .*001	7.25 (5.62–9.34), *P < .*001
Ordinal trend (continuous)	–	1.77 (1.68–1.86), *P < .*001	1.66 (1.57–1.76), *P < .*001

a Adjusted for age, gender identity, ethnicity, household/peer smoking, and family affluence.

Table 3 shows the association between household secondhand smoke exposure and regular e-cigarette use among adolescents who had never smoked cigarettes (*n* = 11 907). A clear dose–response relationship was observed. Compared to those with no reported exposure in the past year, adolescents exposed less than monthly had slightly higher odds of regular e-cigarette use (aOR = 1.39, 95% CI: 1.00–1.95), though this association was marginally non-significant (*P* = .058). More frequent exposure was associated with progressively greater odds of use: 2.47 (95% CI: 1.72–3.55) for those exposed 1–2 times per month, 2.91 (95% CI: 2.02–4.19) for 1–2 times per week, and 5.04 (95% CI: 3.64–6.99) for daily or near-daily exposure (all *P < .*001). The ordinal trend analysis further supported a strong graded relationship (aOR per category = 1.50, 95% CI: 1.39–1.61, *P < .*001). These associations were independent of age, gender identity, ethnicity, household or peer smoking, and family affluence.

**Table 3.** Adjusted association between household secondhand smoke exposure and regular e-cigarette use among adolescents who have never smoked cigarettes (*n* = 11 907)

Secondhand smoke exposure	*N*	Crude OR, 95% Cl, *P* value	Adjusted OR, 95% Cl, *P* value^a^
Never in the past year	4943	Reference	Reference
Less than monthly	2739	1.88 (1.36–2.59), *P < .*001	1.39 (1.00–1.95), *P* = .058
1–2 times per month	1362	3.25 (2.32–4.56), *P < .*001	2.47 (1.72–3.55), *P < .*001
1–2 times per week	1358	3.74 (2.70–5.19), *P < .*001	2.91 (2.02–4.19), *P < .*001
Daily/most days	1505	6.71 (5.04–8.94), *P < .*001	5.04 (3.64–6.99), *P < .*001
Ordinal trend (continuous)	–	1.57 (1.48–1.67), *P < .*001	1.50 (1.39–1.61), *P < .*001

a Adjusted for age, gender identity, ethnicity, household/peer smoking, and family affluence.

## Discussion

This study provides evidence that household exposure to secondhand tobacco smoke is strongly and consistently associated with regular e-cigarette use among adolescents in England. We observed that adolescents who reported being exposed to cigarette smoke in their own or someone else’s home were significantly more likely to report vaping regularly—defined as at least once per week. The association exhibited a strong dose–response gradient across increasing frequencies of secondhand smoke exposure, even after adjusting for age, gender identity, ethnicity, family affluence, and whether anyone close to the adolescent smoked. Those exposed ‘daily or most days’ had over seven times the odds of regular e-cigarette use compared to adolescents who reported no exposure in the past year. This graded pattern was statistically significant in both categorical models and when exposure was treated as a continuous ordinal variable. The robustness of the association across analytic approaches suggests that secondhand smoke exposure may function not only as a marker of a high-risk environment, but also as a psychosocial and behavioural driver of e-cigarette uptake.

A particularly noteworthy finding is that the association between secondhand smoke exposure and regular vaping persisted when the analysis was restricted to adolescents who had never smoked cigarettes. Among this subsample of over 11 000 never-smokers, exposure to secondhand smoke ‘daily or most days’ was associated with a fivefold increase in the odds of regular vaping, compared to their unexposed peers. This suggests that the influence of secondhand smoke exposure extends beyond youth already engaged in tobacco use. It also counters the assumption that e-cigarette uptake in smoke-exposed adolescents is primarily explained by co-use of conventional cigarettes. These findings are consistent with social learning theory, which posits that young people acquire behaviours by observing the actions of role models in their immediate environment [17–19]. Adolescents who regularly observe family members or others smoking may come to view nicotine use as normative, socially acceptable, or even inevitable. The sensory cues of smoke, its smell, visibility, and social context, may also prime curiosity and receptiveness to trying alternative nicotine delivery systems like vaping. The strength of this association among never-smokers reinforces the hypothesis that secondhand smoke exposure plays an independent role in initiating nicotine use via e-cigarettes, not merely reflecting shared household behaviours.

This study extends a growing body of literature that has predominantly focused on peer influence, advertising, or individual-level risk factors for adolescent vaping [6–8, 20]. Although peer dynamics remain a powerful driver of adolescent substance use, our findings highlight the often-overlooked role of the household as a foundational context for exposure and initiation. While England has made significant progress in implementing smoke-free legislation in public places, smoking in private homes remains largely unregulated [21]. As a result, children and adolescents may continue to experience high levels of exposure to secondhand smoke, particularly in households where socioeconomic disadvantage and parental smoking intersect. Importantly, these findings call attention to the need for whole-household interventions—not only to reduce passive smoke exposure but also to shift household norms and expectations regarding all forms of nicotine use. Family-based smoking cessation support, parent-focused education campaigns, and clinical guidance for healthcare providers should explicitly address the risks that secondhand smoke poses beyond physical health, including its potential to catalyse vaping uptake in adolescents.

The public health implications of these findings are substantial. Although most regulatory and educational interventions have focused on limiting the direct availability and marketing of e-cigarettes to young people [22, 23], this study suggests that exposure to secondhand smoke in the home is a powerful environmental factor that may bypass existing regulatory safeguards. By demonstrating that adolescents with greater secondhand smoke exposure are significantly more likely to vape, even if they have never smoked themselves, our findings indicate that a more comprehensive, upstream approach to nicotine prevention is needed. Smoke-free home initiatives should be integrated into existing public health campaigns and school-based health education programmes [24]. Local authorities and NHS services could enhance their impact by promoting voluntary household bans on both smoking and vaping, particularly in homes with children. Moreover, these findings provide a rationale for including questions about secondhand smoke exposure in routine adolescent health assessments, as they may help identify adolescent at elevated risk of nicotine initiation.

This study has several strengths. It is based on a large, nationally representative sample of adolescents aged 11–15 years in England, enhancing the generalizability of the findings to the wider school-aged population. The SDD survey is a well-established instrument, administered under anonymous conditions that reduce the risk of social desirability bias, particularly for sensitive behaviours, such as smoking and vaping. The study benefitted from comprehensive covariate adjustment, including age, gender identity, ethnicity, socioeconomic status, and household or peer smoking status. Furthermore, the use of both categorical and ordinal exposure models, along with subgroup analysis among never-smokers, allowed us to rigorously test the consistency and directionality of the association. Nonetheless, several limitations must be acknowledged. The cross-sectional design precludes definitive conclusions about causality or temporality [25–28]. It is possible that adolescents who already vape are more likely to spend time in environments where smoking occurs, rather than secondhand smoke exposure leading to vaping. These findings should therefore be interpreted as evidence of association rather than causation. Additionally, while our measure of secondhand smoke exposure focused on the home, it did not differentiate between sources (e.g. parents vs. siblings vs. visitors) or include secondhand exposure to vaping aerosols, which are increasingly prevalent. Despite these limitations, the strength and consistency of the associations observed support the conclusion that secondhand smoke exposure remains an important factor for regular adolescent e-cigarette use.

To our knowledge, this is the first nationally representative study in England to assess the association between secondhand smoke exposure and regular (weekly) e-cigarette use among adolescents, using a five-level dose–response measure and stratifying by smoking status. We found a strong, graded association between household secondhand smoke exposure and regular e-cigarette use. Adolescents who were frequently exposed to cigarette smoke in domestic environments were substantially more likely to vape regularly, and this association remained significant even among those who had never smoked conventional cigarettes. Public health campaigns, clinical guidance, and family-based interventions must address the broader social contexts that shape adolescent behaviour, recognizing that the home environment plays a pivotal role in shaping attitudes towards nicotine use from an early age. Tackling secondhand smoke exposure at home may be a key lever in the ongoing effort to curb youth vaping and reduce future nicotine dependence.

## Data Availability

The data analysed in the study is publicly available. NHS England. (2025). Smoking, Drinking and Drug Use among Young People, 2023 [data collection]. UK Data Service. SN: 9366, DOI: http://doi.org/10.5255/UKDA-SN-9366-1. Key points What is known: Adolescent e-cigarette use is increasing, and prior studies have highlighted peer influence and marketing, among others, as major drivers of vaping uptake. What is unknown: The role of household secondhand smoke exposure, particularly its frequency, in shaping adolescent e-cigarette use remains underexplored, especially among youth who have never smoked cigarettes. What this study adds: This study identifies a strong and graded association between household secondhand smoke exposure and regular e-cigarette use among adolescents in England. The relationship persists even among never-smokers, suggesting secondhand smoke exposure may independently increase susceptibility to vaping.
